# Lymphocyte subset analysis to evaluate the prognosis of HIV-negative patients with pneumocystis pneumonia

**DOI:** 10.1186/s12879-021-06124-5

**Published:** 2021-05-14

**Authors:** Fan Jin, Jing Xie, Huan-ling Wang

**Affiliations:** 1 Department of Infectious Diseases, Peking Union Medical College Hospital, Chinese Academy of Medical Sciences & Peking Union Medical College, No.1 Shuaifuyuan, Wangfujing, Dongcheng District, Beijing, 100730 China; 2 Clinical Pharmacology Research Center, Peking Union Medical College Hospital, Chinese Academy of Medical Sciences & Peking Union Medical College, Beijing, 100730 China

## Abstract

**Objectives:**

We analysed the peripheral blood lymphocyte subsets of human immunodeficiency virus (HIV)-negative patients infected with pneumocystis pneumonia (PCP) to determine the relationships between the levels of different types of lymphocytes and the prognosis of patients.

**Methods:**

We retrospectively reviewed HIV-negative patients with PCP diagnosed in our department. All the eligible patients underwent lymphocyte subset analysis on admission.

**Results:**

A total of 88 HIV-negative PCP patients were enrolled in the study. In univariate analyses, low CD4+ T cell count, low CD8+ T cell count, and low natural killer cell (NK cell) count were associated with higher in-hospital mortality. CD8+ T cell count ≤300/μL was found to be an independent risk factor for poor prognosis in multivariate logistical regression analysis (*p* = 0.015, OR = 11.526, 95% CI = 1.597–83.158). Although low CD4+ T cell and NK cell counts were not independent risk factors, the mortality rates of PCP patients decreased as the CD4+ T cell and NK cell counts increased.

**Conclusion:**

The immune process of *Pneumocystis jirovecii* infection is complex but important. We propose that lymphocyte subsets could give clinicians a better understanding of patient immune status, helping with the early identification of potentially lethal infections and treatment decision making, such as adjusting the immunosuppressive regimen and choosing an appropriate patient monitoring level.

## Introduction

Pneumocystis pneumonia (PCP) is a life-threatening interstitial pneumonia caused by *Pneumocystis jirovecii* in immunocompromised patients [1, 2]. PCP has long been known for its high prevalence in acquired immunodeficiency deficiency syndrome (AIDS) patients [3], but the incidence of PCP in human immunodeficiency virus (HIV)-positive patients has been decreased largely due to the broader use of highly active antiretroviral therapy (HAART) and trimethoprim-sulfamethoxazole (TMP/SMZ) prophylaxis when the CD4+ T cell count is < 200/μL [4, 5]. Recently, PCP has been frequently diagnosed in HIV-negative patients, as immunosuppressive regimens are being increasingly used in a wide range of patients. The most common underlying conditions of PCP in non-HIV-infected patients are haematological malignancies, followed by solid tumours, autoimmune diseases, solid organ transplantation, etc. [6, 7]. The mortality rate among patients with PCP in this population is 30–60% [6]. HIV-negative PCP patients are more likely to have acute onset, more severe symptoms, and worse prognosis than HIV-positive patients [6]. Identifying potential prognostic factors in PCP patients could help clinicians choose appropriate treatment strategies and monitoring levels.

The major portion of the PCP process results from the host inflammatory response to apparent infection with *P. jirovecii* rather than direct tissue damage from *P. jirovecii* itself [8, 9]. Because PCP is a type of opportunistic infection (OI), lymphocyte subsets can be used as important indicators of the immune function of the body and can help with the clinical diagnoses of some infectious diseases, which is of great significance for analysing the pathogenesis, observing the curative effect, and predicting the prognosis [10]. T cells are known to be involved in the suppression of the autoimmune response and hyperinflammation mediated by CD4+ T cells [11]. B cells and NK cells are critical for an effective CD4+ T cell response that can control Pneumocystis infection [12]. In this article, we analysed the peripheral blood lymphocyte subsets of HIV-negative PCP patients to determine the influence of T lymphocyte, B lymphocyte and natural killer cell (NK cell) counts on prognosis.

## Material and methods

### Patients selection

We carried out an observational retrospective study from 2012 to 2018 in the Peking Union Medical College Hospital (PUMCH), a 2000-bed tertiary care centre in Beijing, China. PCP was diagnosed based on consensus guidelines, which require either *P. jirovecii* microorganisms in respiratory samples by microscopic examination or both a positive polymerase chain reaction (PCR) test for *P. jirovecii* DNA in respiratory samples and an increased level of serum 1,3-β-D-glucan (βDG) [13]. Patients were eligible for enrolment if they fulfilled all the following criteria: (1) being older than 18 years of age; (2) being HIV negative; and (3) undergoing lymphocyte subset analysis after presenting with infection on admission. The follow-up time was more than 3 months. We excluded patients who were lost to follow-up or had incomplete data.

This retrospective study was reviewed and approved by the Institutional Review Board (IRB) of PUMCH. Researchers did not interfere with the treatment plan of patients, and they prevented the information of patients from being disclosed. The study met the IRB’s minimal risk waiver criteria; therefore, the requirement to obtain informed consent from each patient was waived.

### Technical information

We used primers to amplify the major surface glycoprotein (MSG) gene of *P. jirovecii* by PCR. The primer sequences for amplifying MSG were MSG-fw, 5′-CTTAAAATAAATAATCAGACTATGTGCGATAAG-3′, and MSG-rv, 5′-GGAGCTTTAATTACTTTTTTCTGGC-3′. A dual-labelled fluorescence resonance energy transfer (FRET) hybridization probe (MSG-probe 5′-FAM-TAGATAGTCGAAAGGGAAA-MGE-3′) was used for detection. The cycle threshold (Ct) value was checked for all positive samples. This was defined as the replication cycle number at which the fluorescence generated within a reaction crossed the fluorescence threshold. A lower Ct value correlates with a higher fungal burden. According to a previous study, a qPCR Ct value above 35 correlates with a clinically low fungal burden and negligible colonization [14]. A positive qPCR result in our study was defined as a Ct value ≤37.

Lymphocyte subset analysis was performed in the clinical haematology laboratory by trained personnel using a FACScan flow cytometer (Beckman-Coulter, USA). The monoclonal antibodies used in this study (purchased from Beckman-Coulter and Immunotech, USA) included PEcy5-CD4/PE- CD8/FITC-CD3 (CD4+/CD8 + T cell count) and PEcy5-CD19/PE- CD16 CD56/FITC-CD3 (B/NK cell count).

### Data collection

All medical records were reviewed retrospectively using a standardized research protocol. The chart review included demographic characteristics, underlying diseases, laboratory data, presence of co-infection, immunosuppressive treatments, the dosage and exposure time of corticosteroid (CS) treatment, and the time from symptom onset to anti-PCP treatments. Additionally, the need for intensive care unit (ICU) admission and mechanical ventilation (MV) and the clinical outcomes were recorded for all patients. Co-infections, such as with bacteria, *Candida*, *Aspergillus*, *cytomegalovirus*, and Epstein-Barr virus, were also evaluated. Survival was defined as being alive 3 months after symptom onset.

### Statistical analysis

Categorical variables were analysed by the chi-square test or Fisher’s exact test, as appropriate. Continuous variables were analysed using the Wilcoxon signed-rank test. In order to determine the best critical threshold of these cell counts for predicting the prognosis, we constructed receiver operating characteristic (ROC) curves to investigate the predictors of in-hospital mortality. The ROC curve showed the tradeoff between true detections and false detections in univariate analysis in predicting the prognosis of PCP. On each ROC curve, we calculated the point with the largest Youden index (Youden index = sensitivity + specificity − 1) as the cut-off threshold. To identify predictors of in-hospital mortality or survival, we applied univariate and multivariate logistical regression analyses and Cox regression hazards models. Consequently, both odds ratio (OR) and hazard ratio (HR) estimates are reported. The 95% confidence intervals (CIs) were calculated. In addition, we calculated the time from symptom onset to all-cause mortality from multivariable Cox regression models. All statistical analyses were performed using SPSS 22.0 (SPSS Inc., Chicago, IL, USA). *P*-values < 0.05 were considered statistically significant.

## Results

### Baseline patient characteristics

A total of 180 HIV-negative patients had positive PCR and/or Grocott’s methenamine silver (GMS) staining results on sputum or bronchoalveolar lavage (BAL). A total of 88 patients met the inclusion criteria (Table 1). The median age of the patients was 57 years old, and 42% of them were male. The most common underlying disease was autoimmune disease (*n* = 61, 69%); others included haematological disorders, haematologic malignancies, solid cancers, severe combined immunodeficiency, idiopathic pulmonary fibrosis, and solid organ transplantation. Thirty-four percent of the patients had underlying lung diseases, such as interstitial lung disease caused by autoimmune diseases, lung metastasis caused by solid tumours, and idiopathic pulmonary fibrosis. Immunosuppressive regimens consisted mainly of a combination of CS and immunosuppressive drugs. The median CS exposure time of patients was 3 months (interquartile range 2 to 42 months). The majority of patients were complicated with viral infection (63%), followed by bacterial (30%) and fungal infections (18%). Compared with the normal ranges, patients with PCP had significantly low immune cell counts (including CD19+ B cells, CD16 + CD56+ NK cells, and CD3+ T cells) and a low ratio of CD4+/CD8+ cells (median ratio was 0.6).

**Table 1:** Clinical characteristics, management, and outcomes of in HIV negative patients with PJP

	Total (*n* = 88)	Survivors (*n* = 49)	Non-survivors (n = 39)	P-value
Demographics				
Age, years	58 (42–64)	58 (48–64)	55 (40–63)	0.559
Sex (male)	37 (42%)	22 (45%)	15 (39%)	0.543
Underlying lung disease	30 (34%)	12 (24%)	18 (49%)	0.033
Immunosuppressive conditions				
Autoimmune diseases	61 (69%)	32 (66%)	29 (74%)	0.360
Hematologic disorder	6 (7%)	4 (8%)	2 (5%)	0.609
Hematologic malignancy	5 (6%)	1 (2%)	4 (10%)	0.098
Solid cancer	8 (9%)	5 (10%)	3 (8%)	0.684
Other disease^a^	8 (9%)	7 (14%)	1 (3%)	0.057
Laboratory values on admission				
Albumin, g/L	27 (24–31)	29 (25–32)	26 (24–29)	0.027
LDH, U/L	566 (422–718)	460 (332–612)	656 (557–946)	< 0.001
PaO2, mmHg	53 (46–59)	56 (47–61)	51 (39–59)	0.174
D(A-a)O2, mmHg	60 (52–68)	60 (53–66)	61 (52–69)	0.157
Creatinine, mg/d	68 (53–110)	65 (53–94)	69 (54–115)	0.146
CRP, mg/dL	12 (6–80)	9 (5–63)	21 (6–114)	0.629
Flow cytometry on admission^b^				
Lymphocyte count (× 106 g/L)	360 (237–711)	499 (280–1032)	301 (160–406)	0.162
CD19+ B, cell/μL	36 (11–102)	55 (18–141)	34 (8–64)	0.346
CD16 + CD56+ NK, cell/μL	34 (14–73)	50 (26–83)	22 (7–46)	0.014
CD3+ T, cell/μL	297 (174–662)	517 (234–871)	209 (138–330)	< 0.001
CD4+ T, cell/μL	120 (48–232)	149 (82–314)	81 (35–145)	0.004
CD8+ T, cell/μL	151 (78–356)	239 (101–455)	114 (59–204)	< 0.001
CD4+/CD8+ ratio	0.53 (0.31–1.38)	0.6 (0.3–1.4)	0.6 (0.4–1.5)	0.971
Immuosuppressive conditions				
CS exposure time, month	3 (2–42)	4 (2–22)	3 (2–53)	0.490
CS ≤ 40 mg/d	56 (64%)	30 (61%)	26 (67%)	0.598
500 mg/d > CS ≥ 80 mg/d	5 (6%)	2 (4%)	3 (8%)	0.467
CS ≥ 500 mg/d	8 (9%)	2 (4%)	6 (15%)	0.067
Non-steroidal immunosuppressive therapy				
Cyclophosphamide	33 (38%)	17 (35%)	16 (41%)	0.542
Methotrexate	9 (10%)	4 (8%)	5 (13%)	0.474
Mycophenolate mofetil	8 (9%)	6 (12%)	2 (5%)	0.249
Azathioprine	2 (2%)	1 (2%)	1 (3%)	0.870
Leflunomide	8 (9%)	4 (8%)	4 (10%)	0.734
Tacrolimus	5 (6%)	2 (4%)	3 (8%)	0.467
CS + immunosuppressive drug	52 (59%)	27 (55%)	25 (64%)	0.394
Chemotherapy	14 (16%)	6 (12%)	8 (21%)	0.292
Imaging manifestations				
Ground glass opacity	69 (78%)	38 (79%)	31 (84%)	0.826
Consolidations	20 (23%)	10 (21%)	10 (27%)	0.561
Co-infections				
Bacterial	26 (30%)	13 (27%)	12 (31%)	0.661
Viral	55 (63%)	30 (61%)	25 (64%)	0.780
Fungal	16 (18%)	10 (20%)	6 (15%)	0.544
Clinical progression				
Symptoms onset to treatment, days	7 (4–15)	8 (5–16)	7 (3–12)	0.359
Mechanical ventilation	44 (50%)	10 (20%)	34 (87%)	< 0.001

^a^Others: Severe combined immunodeficiency, idiopathic pulmonary fibrosis and solid organ transplantation

^b^Normal reference value: CD19+ B 180–324 cell/μL, CD16 + CD56+ NK 175–567 cell/μL, CD3+ T 1185–1901 cell/μL, CD4+ T 561–1137 cell/μL, CD8+ T 404–754 cell/μL, and CD4+/CD8+ ratio 0.95–2.13

Data are presented as median (interquartile range) and n (%)

### Clinical parameters and outcomes

As shown in Tables 1, 44% (*n* = 39) of HIV-negative patients infected with PCP died within 3 months after symptom onset. No differences were noted in age, sex, previous use of chemotherapy, immunosuppressive regimen, dosage or exposure time of CS treatment, co-infections (bacterial, viral, or fungal), or treatment delay (symptom onset to treatment) between survivors and non-survivors. Compared with survivors, non-survivors had significantly lower albumin levels (*p* = 0.027), higher LDH levels (*p* < 0.001), and higher demand for mechanical ventilation (*p* < 0.001). More non-survivors had underlying lung diseases (*p* = 0.033). As for lymphocyte subsets, non-survivors had a significantly lower NK cell count (22 vs. 50 cell/μL, *p* = 0.014), lower CD4+ T cell count (81 vs. 149 cell/μL, *p* = 0.004) and lower CD8+ T cell count (114 vs. 239 cell/μL, *p* < 0.001). There was no significant difference in B cell count between survivors and non-survivors (55 vs. 34 cell/μL, *p* = 0.346).

### Lymphocyte subset analysis and prognosis

**Fig. 1:**
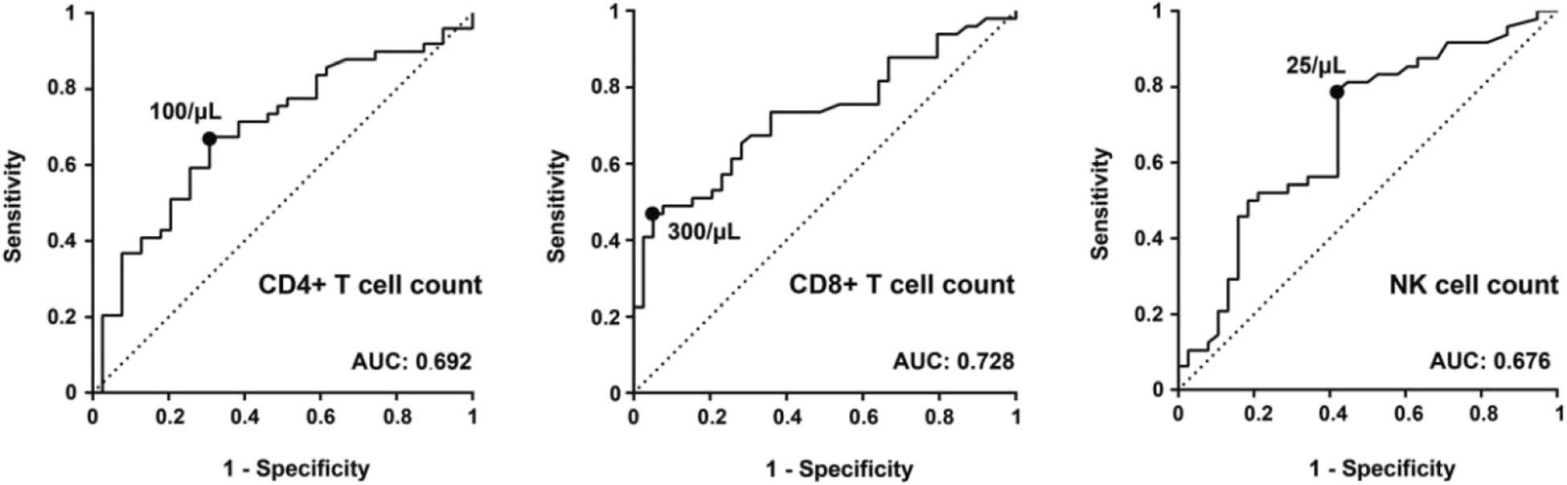
ROC curves for the predictors of in-hospital mortality for CD4+ T, CD8+ T, and NK cell counts

Compared with survivors in the univariate analysis, the non-survivors had lower CD4+ T, CD8+ T, and NK cell counts (Table 2). As shown in Fig. 1, the area under the curve (AUC) of CD8+ T cell count was 0.728 (*p* < 0.001), indicating that CD8+ T cell count was an important predictor of in-hospital mortality. The optimal cut-off for CD8+ T cell count was determined to be 300/μL, and the specificity and sensitivity were 0.95 and 0.47, respectively. The area under the CD4+ T cell count curve (*p* = 0.020) and the area under the NK cell count curve (*p* = 0.005) were slightly smaller. The optimal cut-off for CD4+ T cell count was determined to be 100/μL, with a specificity and sensitivity of 0.71 and 0.67, respectively. The optimal cut-off for NK cell count was determined to 25/μL, with a specificity and sensitivity of 0.68 and 0.79.

**Table 2:** Characteristics associated with mortality in a univariate and multivariate analysis in HIV negative patients with PJP

Characteristics	Univariate analysis		Multivariate analysis	
	OR (95% CI)	*P*-value	OR (95% CI)	*P*-value
Underlying lung disease				
no	1		1	
yes	2.643 (1.068–6.538)	0.035	2.134 (0.501–9.089)	0.305
Albumin				
≥ 30	1			
< 30	2.500 (0.981–6.371)	0.055		
LDH, U/L				
< 600	1			
≥ 600	2.196 (0.928–5.198)	0.074		
CD4+ T, cell/μL				
≥ 100	1		1	
< 100	3.793 (1.341–10.729)	0.012	0.255 (0.040–1.646)	0.151
CD8+ T, cell/μL				
≥ 300	1		1	
< 300	10.615 (2.880–39.130)	< 0.001	11.526 (1.597–83.158)	0.015
CD16 + CD56+ NK, cell/μL				
≥ 25	1		1	
< 25	3.990 (1.609–9.893)	0.003	2.168 (0.561–8.379)	0.262
Mechanical ventilation				
no	1		1	
yes	26.520 (8.248–85.265)	< 0.001	33.578 (8.056–139.950)	< 0.001

**Fig. 2:**
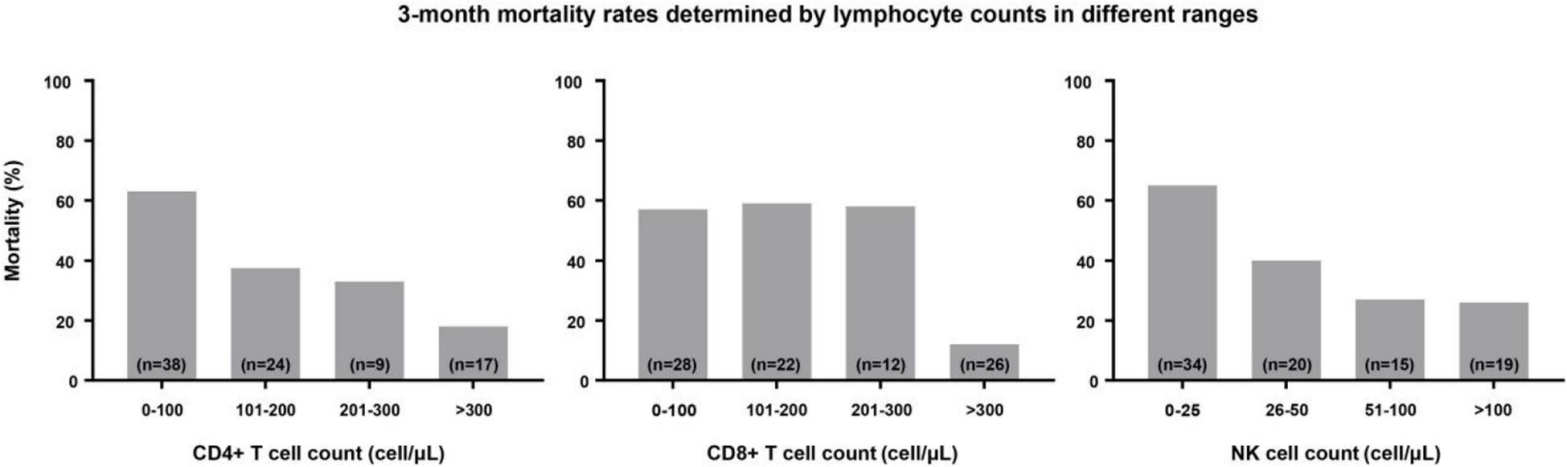
The 3-month mortality rates determined according to the levels of CD4+ T, CD8+ T, and NK cell counts over different ranges

The 3-month mortality rates of patients with different CD4+ T, CD8+ T, and NK cell counts are shown in Fig. 2. We found that the mortality rates of patients decreased sequentially in the four CD4+ T cell count ranges of 0–100/μL, 101–200/μL, 201–300/μL, and > 300/μL, being equal to 63, 38, 33 and 18%, respectively. Similarly, the mortality rates of patients with NK cell counts of 0–25/μL, 26–50/μL, 51–100/μL, and > 100/μL were 65, 40, 27 and 26%. The mortality rates were almost the same between patients with CD8+ T cell counts of 0–100/μL, 101–200/μL, and 201–300/μL, but for patients with CD8+ T cell count > 300/μL, the mortality rate dropped by more than 40%.

### Independent predictors of mortality in PCP patients

Prognostic factors of 3-month mortality were explored through logistic regression. Table 2 shows the results of the multivariate analysis. In the multivariate analysis, corrections were made for underlying lung disease, need for mechanical ventilation, and CD4+ T, CD8+ T, and NK cell counts. In HIV-negative patients with PCP, the presence of a CD8+ T cell count ≤300/μL (*p* = 0.015, OR = 11.526, 95% CI = 1.597–83.158) and a demand for mechanical ventilation (*p* < 0.001, OR = 33.578, 95% CI = 8.056–139.950) were independent predictors of 3-month all-cause mortality.

**Fig. 3:**
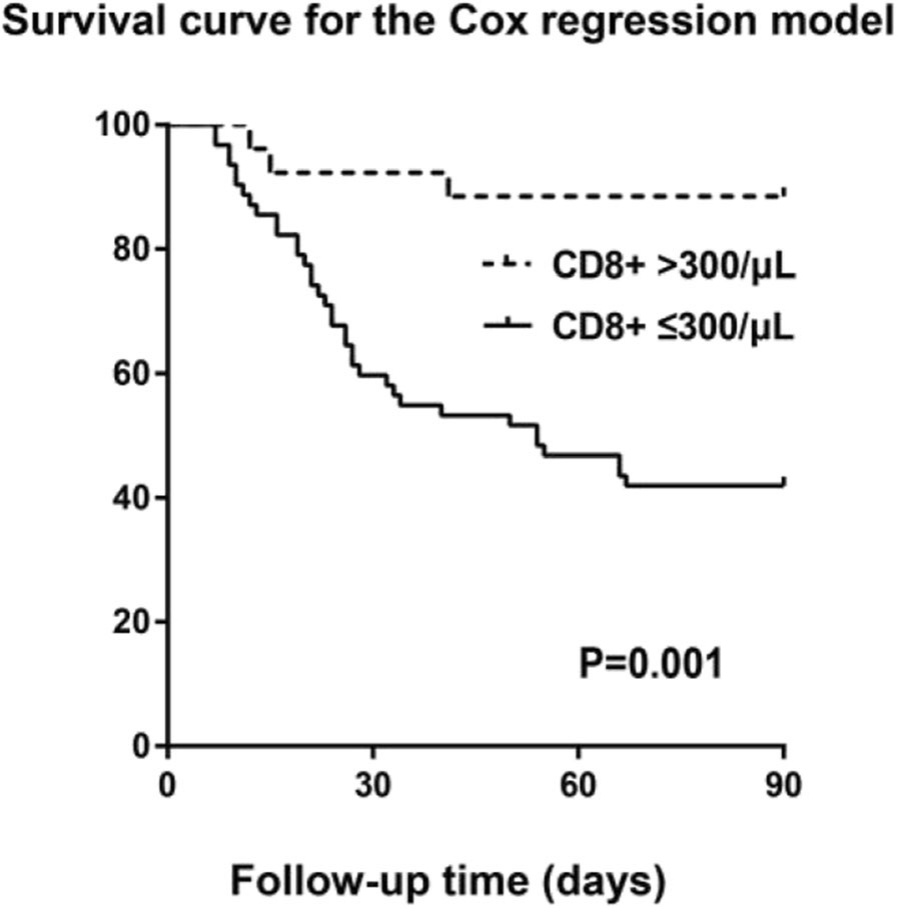
Survival curve for the Cox regression hazards model for the relationship between CD8+ T cell count and 3-month mortality

We next performed a Cox regression hazards model to further understand the interdependence of CD8+ T cell count as a determinant of 3-month mortality. The survival probability curve is shown in Fig. 3. The results further confirmed that a low CD8+ T cell count was significantly associated with high mortality (*p* = 0.001, HR = 6.799, 95% CI = 2.090–22.121).

## Discussion

In this study, all 88 HIV-negative patients with PCP had long-term and varying degrees of immunosuppression, mostly manifesting as diffuse lymphocyte depletion. Dichotomizing patients around the threshold calculated from ROC analysis, we identified a CD8+ T cell count < 300/μL as an independent risk factor for death in PCP patients. Additionally, the HR of CD8+ T cell count < 300/μL was 6.799, which was associated with 3-month mortality. Although low CD4+ T cell and NK cell counts were not independent risk factors for death, the mortality rates of PCP patients decreased sequentially with increasing CD4+ T cell and NK cell counts.

In the HIV epidemic of the 1980s, PCP was known as the most common OI among AIDS patients. In recent years, immunosuppressive therapy and biological agents, especially when used in combination, have increased the risk of infection by opportunistic pathogens such as *P. jiroveci* [15]*.* Many large retrospective cohort studies have shown that a low CD4+ T cell count is a risk factor for death in HIV-positive patients infected with PCP [16–20], and lymphopenia is a poor prognostic factor in HIV-negative patients with PCP infection [21]. In animals without HIV infection, an inverse relationship between the *P. carinii* burden and the level of circulating CD4+ or CD8+ T lymphocytes has been found [22], which demonstrated the impact of immune system status on susceptibility to opportunistic pathogens. However, the prognostic value of lymphocyte subsets in HIV-negative PCP patients remains unclear. One small retrospective study revealed that CD4+ T cell count < 200/μL in kidney transplant recipients was a poor prognostic factor for PCP infection [23], and another showed that CD8+ T cell count < 160/μL was strongly associated with mortality in autoimmune disease patients with PCP [24]. However, we identified CD8+ T cell count < 300/μL as an independent risk factor for death in HIV-negative PCP patients. In addition, NK cell count was included in such an analysis for the first time.

As a threshold for predicting mortality, the sensitivity of CD8+ T cell count < 300/μL was low in our analysis. This means that other values below this threshold may also be correlated with poor prognosis. As shown in Fig. 2, the mortality rates of patients with CD8+ T cell counts ranging from 0 to 100/μL and 101–200/μL were very close to that of the 201–300/μL group. The mortality of the patients was significantly reduced when the CD8+ T cell count was > 300/μL, which demonstrates the high specificity of this threshold. We suggest paying more attention to patients with CD8+ T cell counts below 300/μL.

The pathogenesis and prognosis of opportunistic infection are closely related to immune status, and the importance of lymphocytes in combatting *P. jirovecii* infection has been recognized. Lymphocytes participate in host defence by regulating other immune cells, producing antibodies against pathogens (B cells) and killing organisms (cytotoxic CD8+ T cells and NK cells) [25]. CD4+ T cells can promote the proliferation and differentiation of B cells, T cells, and other immune cells; coordinate the interaction between immune cells; and play a central role in coordinating host defence mechanisms. CD8+ T cells include inhibitory T cells and killer T cells [26]. If the inflammatory response is driven by CD4+ T cells without sufficient inhibitory activity from CD8+ T cells, it will cause an effective but excessive inflammatory response similar to immune restoration disease (IRD), and the elimination of pathogens will incur severe lung injury as a cost [27]. Dysregulation of CD4+ T cells can lead to a decrease in their ability to assist CD8+ T cells, which in turn leads to a reduction in the killing and dissolving function of CD8+ T cells; that is, the regulation of the immune response becomes imbalanced in the negative direction. In our study, a CD8+ T cell count < 300/μL was an independent risk factor for death in PCP patients. Although CD4+ T cells play a central role in coordinating host defences against *P. jirovecii*, the inhibition and cytotoxic effects of CD8+ T cells seem to be critical to control and terminate the infection.

The NK cell count was also associated with the prognosis of PCP patients. Most striking of our findings was the progressive increase in mortality as NK cell count decreased from > 100/μL (26% mortality) to < 25/μL (65% mortality). NK cells play an important role in the immune defence against *P. jirovecii* infection by releasing IFN-γ, granzyme, and perforin and by their direct microbial activity [28, 29]. Recent studies have demonstrated that NK cells directly regulate adaptive immune responses through their interaction with CD4+ T cells [30–32]. Therefore, we believe that the NK cell count has certain prognostic value for PCP patients. Being aware of the importance of NK cells in combatting *P. jirovecii* infection, it is necessary for us to further clarify the immune process in response to *P. jirovecii* infection in humans.

We usually believe that the longer the exposure time and the higher the dose of immunosuppressive therapy, the more severe the degree of immunosuppression is. However, in our study, no association was found between the prognosis of PCP and the type of immunosuppressive regimen, the dosage, or the exposure time. The reason for this lack of correlation may be that the number of patients taking the different immunosuppressive regimens was very small, so the fact that almost all patients received high-dose immunosuppressive therapy eliminated the distinction between low and high levels of immunosuppression. The findings also imply that the prognosis of PCP patients cannot be simply reflected by their immunosuppressive therapy. Our study revealed that low CD4+ T cell, CD8+ T cell, and NK cell counts were correlated with a poor prognosis of PCP, especially CD8+ T cell counts. Therefore, for patients receiving high-dose immunosuppressive therapy, lymphocyte subset analysis can greatly help clinicians assess the degree of immunosuppression. In some studies, CD4+ T cell count < 200/mm^3^ was taken as the indicator of a prevention strategy of PCP in HIV-negative patients, following the practice in HIV-positive patients [33]. Our study demonstrates that several patients have CD4+ T > 200/mm^3^, and CD8+ T and NK cells are also associated with the prognosis of these patients. Because there are a variety of changes in the immune system in HIV-negative patients, they cannot be described by the CD4+ T cell count alone. Therefore, the CD4+ T cell count should not be the only indication of prophylaxis in HIV-negative patients, and the levels of CD8+ T and NK cell counts should also be considered.

For HIV-positive patients with severe PCP, a large amount of medical-based evidence has shown that CSs as an adjuvant treatment can reduce mortality [34]. However, for HIV-negative PCP patients, due to the non-specific anti-inflammatory effects and serious side effects of CSs, the application of CSs in immunocompromised individuals is still controversial [35]. Therefore, alternative treatments for HIV-negative patients with PCP need to be more specific. Recent studies have suggested that CD4 + CD25+ T cells [36] and CD40 ligand [30] seem to be helpful in the regulation of immunity to *P. jirovecii* infection*.* CD4 + CD25+ T cells seem to suppress proliferation and cytokine production to limit the occurrence of immune reconstitution disease [37]. CD40L+ effector memory CD4+ T cells can control the reactivation and maintain the response of NK cells through IL-2 production and can promote the secretion of chemokine ligand-10 (CXCL10) from dendritic cells [38]. In a murine experimental model, CD40 ligand [39–41] and anti-CD3 monoclonal antibody [42] have been confirmed to improve the prognosis of PCP infection. For HIV-negative patients with PCP, in addition to extensive antifungal therapy, adjusting immune disorders and attenuating excessive inflammatory responses may be a new treatment strategy.

There were several limitations to this study. First, it was a single-site study. Second, autoimmune diseases were the predominant underlying conditions among our study patients. Studies of HIV-negative PCP patients with other underlying disorders, such as organ transplantation, may yield different findings. Third, we only analysed the lymphocyte subsets of the patients after onset of the infection, but baseline lymphocyte subsets, which may also have an influence on mortality prediction, were unknown. A prospective study may be meaningful for further analysis. Fourth, follow-up results beyond 3 months were not available, so we do not know the long-term outcome of this patient cohort. Our study provides evidence of the correlation between lymphocyte subsets and short-term prognosis among HIV-negative PCP patients. Our findings suggest that a lower CD8+ T cell count is an independent risk factor for mortality, and in addition to effective anti-fungal therapy, treatments aimed at regulating immune function should be investigated.

## Conclusion

In conclusion, the immune process of *P. jirovecii* infection in the human body is complex but important. Low CD4+ T cell, CD8+ T cell and NK cell counts were related to the poor prognosis of PCP in HIV-negative patients, and a CD8+ T cell count below 300/μL was an independent predictor for death. Thus, we proposed that clinicians can use lymphocyte subsets to identify potential patients and make clinical decisions, such as adjusting the immunosuppressive regimen and choosing an appropriate patient monitoring level.

## Data Availability

The datasets used and/or analysed during the current study are available from the corresponding author on reasonable request.

## Abbreviations

BAL: Bronchoalveolar lavage
CI: Confidence intervals
Ct: Cycle threshold
D(A-a)O2: Alveolar-arterial oxygen difference
FRET: Fluorescence resonance energy transfer
GMS staining: Grocott’s methenamine silver staining
CXCL 10: Chemokine ligand-10
CS: Corticosteroid
HAART: Highly active antiretroviral therapy
HIV: Human immunodeficiency virus
HR: Hazard ratio
ICU: Intensive care unit
IRB: Institutional review board
IRD: Immune restoration disease
LDH: Lactate dehydrogenase
MV: Mechanical ventilation
MSG: Major surface glycoprotein
NK cells: Natural killer cells
OIs: Opportunistic infections
OR: Odds ratio
PaO2: Arterial partial pressure of oxygen
*P. jirovecii*: *Pneumocystis jirovecii*
PCR: Polymerase chain reaction
PCP: Pneumocystis peumonia
TMP/SMZ: Trimethoprim-sulfamethoxazole
βDG: 1,3-β-D-glucan
